# Hipk is required for JAK/STAT activity during development and tumorigenesis

**DOI:** 10.1371/journal.pone.0226856

**Published:** 2019-12-31

**Authors:** Gritta Tettweiler, Jessica A. Blaquiere, Nathan B. Wray, Esther M. Verheyen

**Affiliations:** Department of Molecular Biology and Biochemistry, Centre for Cell Biology, Development and Disease, Simon Fraser University, Burnaby, B.C Canada; National Cancer Institute, UNITED STATES

## Abstract

*Drosophila* has been instrumental as a model system in studying signal transduction and revealing molecular functions in development and human diseases. A point mutation in the Drosophila Janus kinase JAK (called *hop*) causes constitutive activation of the JAK/STAT pathway. We provide robust genetic evidence that the Homeodomain interacting protein kinase (Hipk) is required for endogenous JAK/STAT activity. Overexpression of Hipk can phenocopy the effects of overactive JAK/STAT mutations and lead to melanized tumors, and loss of Hipk can suppress the effects of hyperactive JAK/STAT. Further, the loss of the pathway effector Stat92E can suppress Hipk induced overgrowth. Interaction studies show that Hipk can physically interact with Stat92E and regulate Stat92E subcellular localization. Together our results show that Hipk is a novel factor required for effective JAK/STAT signaling.

## Introduction

The model organism *Drosophila melanogaster* is a useful tool to study evolutionarily conserved signaling pathways that are used reiteratively during development, as well as for modeling human diseases. The conserved JAK/STAT signalling cascade affects numerous fundamental developmental events, such as oogenesis, embryogenesis, and hematopoiesis. (for review, [1–4]. Dysregulation of the JAK/STAT pathway has been linked to leukemia, myeloproliferative neoplasms, and solid tumors in flies and vertebrates [5–9].

The simplified *Drosophila* JAK/STAT pathway consists of fewer proteins than in mammals, facilitating genetic interaction studies by avoiding genetic redundancy. The core components of the pathway include three ligands Unpaired (Upd/outstretched, Upd2, Upd3), the Domeless receptor (Dome), a single Janus Kinase (JAK) homolog Hopscotch (Hop), and a single STAT homologue, the transcription factor Stat92E [10]. Upon cascade stimulation, Stat92E becomes phosphorylated by Hop, dimerizes, and localizes to the nucleus to regulate JAK/STAT target genes. JAK/STAT mutations in humans are heavily correlated with tumor invasiveness and lethality [11]. *hop*^*Tum-l*^ is a dominant mutation resulting in a hyperactive Hop kinase that leads to constitutive activation of the pathway [12]. Similar activating JAK2 mutations are commonly seen in vertebrate cancers [7,13].

Homeodomain-interacting protein kinase (Hipk in Drosophila, Hipk1-4 in vertebrates) regulates numerous conserved signaling pathways [14–20]. Furthermore, Hipk overexpression can cause hemocyte-derived melanotic tumors similar to those seen in *hop*^*Tum-l*^ flies [21], prompting our investigation into Hipk’s potential role in the JAK/STAT pathway. We find that reduced *hipk* suppressed the severity of *hop*^*Tum-l*^ phenotypes. Further, we provide evidence that Hipk cell-autonomously promotes JAK/STAT signalling *in vivo* and interacts with Stat92E. Our data indicate a novel role for Hipk in modulating JAK/STAT activity.

## Materials and methods

### Genetic crosses and fly stocks

Flies were raised on standard media. Crosses were raised at 25°C unless otherwise noted. *10xstat92E-GFP* (BL#26197) [22], *UAS-eGFP* (BL#5430), *UAS-eGFP* (BL#5431), *hsflp*^*122*^*;;Ubi-RFP*,*FRT79* (made from BL#34498), *y*^*1*^*v*^*1*^*hop*^*Tum*^*/FM7c* (BL#8492; referred to as *hop*^*Tum-l*^), *act5c-GAL4/CyO* (BL#4414), *UAS-MYR-RFP/CyO* (BL#7118), *hml-GAL4* (BL#30139) and *stat*^*06346*^ (BL# 11681) were obtained from Bloomington Drosophila Stock Center, Bloomington, IN. *UAS-hipk*^*RNAi*^ (VDRC ID#108254, [23]) was obtained from Vienna Drosophila Resource Center, Vienna, Austria. Also used were *dpp-GAL4/TM6B* [24], *os*,*y* (a gift from Norbert Perrimon), *UAS-Stat92E-GFP/Cyo* and *UAS-Stat92E-MYC/Cyo*,*wg-lacZ* (a gift from James Castelli-Gair Hombria, [25,26], *PD-lacZ* (a gift from Henry Sun; referred to as *upd*^*1*^*-lacZ* hereon after, Tsai and Sun, 2004 [27]), *ywhsflp*,*tub-GAL4*,*UAS-GFP*,*6X MYC-NLS; UAS-y+;tub-GAL80*,*FRT2A/TM6B* (a gift from Gary Struhl), *ywhsflp*^*122*^*;sp/Cyo;TM2/TM6B*, *UAS-HA-hipk*^*1M*^, *UAS-HA-hipk*^*3M*^, *hipk*^*4*^,*FRT79/TM6B* [28], *UAS-HA-hipk*^*WT*^*-attP40*, *UAS-MYR-HA-hipk-attP40*, *UAS-NLS-HA-hipk-attP40* (made in this study). *act5c-GAL4/Cyo* and *UAS-MYR-RFP/CyO* were recombined to generate *act5c-GAL4*, *UAS-MYR-RFP/CyO*. *hipk*^*4*^, *FRT79/TM6B* and *10xstat92E-GFP/TM6B* were recombined to generate *hipk*^*4*^, *FRT79*,*10xstat92E-GFP/TM6B*.

### Generation of transgenic fly stocks

DNA cloning was performed by the SFU Molecular Biology Service Centre. pCMV-HA-Hipk [28] was used as the source of HA-Hipk. pCMV-MYR-HA-Hipk was created by adding a Src myristoylation (MYR) tag GNKCCSKRQ, [29] before the HA-tag on the N-terminus of Hipk. pCMV-NLS-HA-Hipk was created by adding a SV40 nuclear localization sequence PPKKKRKV [30] before the HA-tag on the N-terminus of Hipk. The EcoRI site of pUASt-attB [31] was mutated to a SmaI site, and HA-Hipk^WT^, MYR-HA-Hipk, and NLS-HA-Hipk were inserted into this site. All constructs were inserted into the attP40 locus generating the fly strains *UAS-HA-Hipk*^*WT*^*-attP40*, *UAS-MYR-HA-hipk-attP40*, and *UAS-NLS-HA-hipk-attP40* (Best Gene, Chino Hills, CA).

### Clonal analysis

Somatic clones were generated by crossing *hsflp*^*122*^*;;Ubi-RFP*,*FRT79* to either *10XStat92E-GFP;hipk*^*4*^,*FRT79/TM6B*, or *upd-lacZ;;hipk*^*4*^,*FRT79/TM6B*. Progeny were heat shocked at 38°C, 48 hours after egg laying for 90 min. MARCM clones were generated by crossing *ywhsflp*^*122*^*;act5c-GAL4*,*UAS-MYR-RFP/CyO;tub-GAL80*,*FRT2A/TM6B* (RFP MARCM79) to either *hipk*^*4*^,*FRT79*,*10xstat92E-GFP/TM6B*, *UAS-HA-hipk*^*WT*^*-attP40;hipk*^*4*^,*FRT79*,*10xstat92E-GFP/SM6a~TM6B*. Progeny were heat shocked at 38°C, 48 hours after egg laying for 90 minutes and were subsequently raised at 29°C.

### Immunocytochemistry and microscopy

Third instar larval (L3) imaginal discs were dissected and stained using standard protocols [21]. The following primary antibodies were used: rabbit anti-Hipk 9744 (1:200; generated in our lab), rat anti-Ci (1:20; 2A1 DSHB), mouse anti-En (1:10; 4D9 DSHB), chicken anti-beta galactosidase (1:1000; Abcam), rabbit anti-β galactosidase (1:800; Cappell), mouse anti-HA (1:200; ABM), rabbit anti-HA (1:2000; Cell signaling), mouse anti-MYC (1:200; ABM). The following secondary antibodies were obtained from Jackson Immunoresearch (all 1:300): DyLight649 anti-rabbit, DyLight649 anti-mouse, and Cy3 anti-rabbit, FITC anti-chicken. Nuclei were detected by staining with DAPI. Immunofluorescent images were acquired using a Nikon Air laser-scanning confocal microscope. Adult flies and pupae were imaged with a Canon Rebel T1i while submerged in ethanol. Images were processed with Nikon Elements, Adobe Photoshop, and Helicon Focus. For a subset of fluorescent images channel colours were converted to accommodate colour blind viewers.

### *hop*^*Tum-l*^ lethality and tumor frequency assays

**Fig 1:**
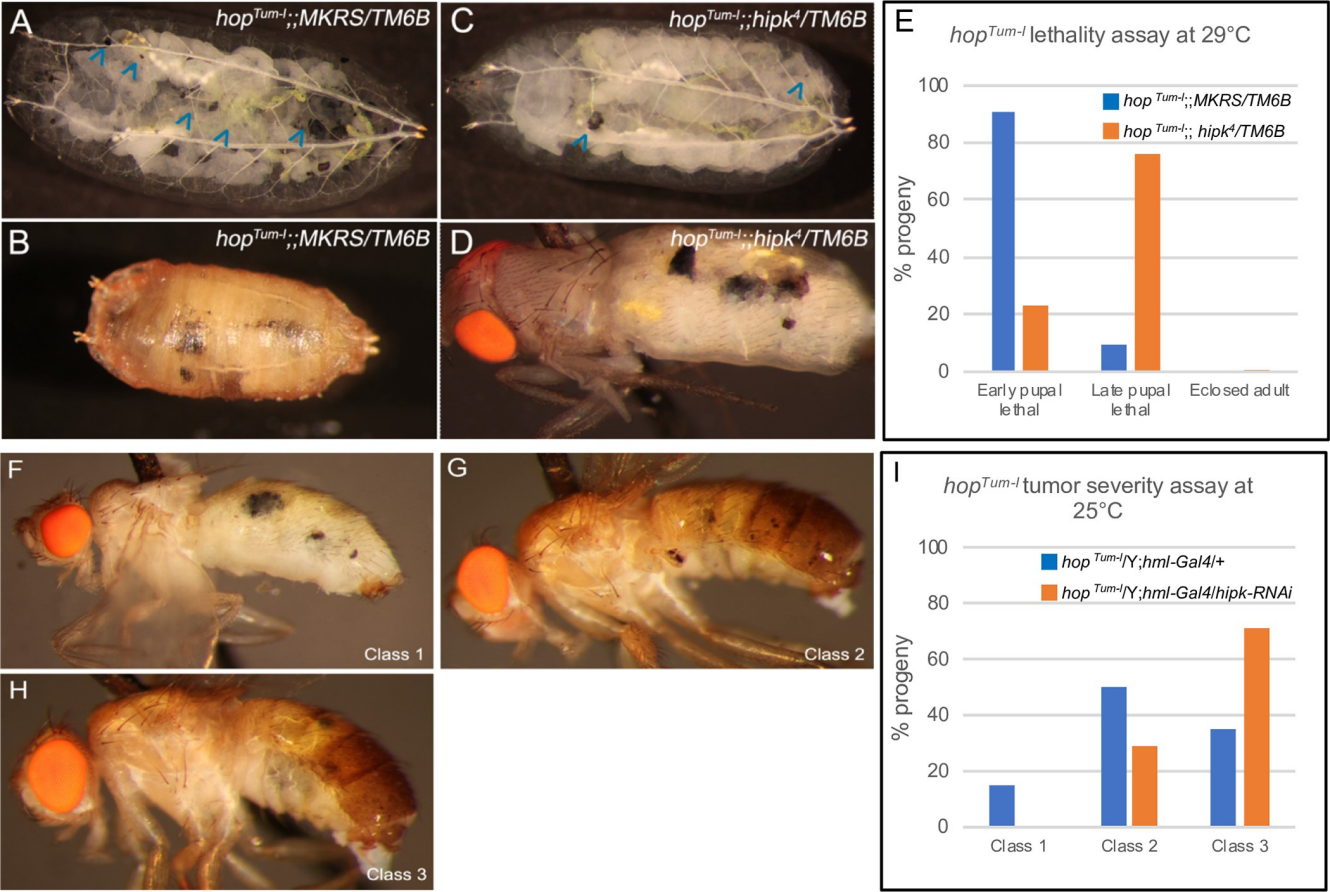
Heterozygous loss of *hipk* suppresses *hop*^*Tum-l*^ induced lethality and tumor load. (A, B) At 29°C *hop*^*Tum-l*^ causes the formation of melanized tumors (A; arrowheads) and (B) results in larval and/or pupal lethality. (C, D) Heterozygous loss of *hipk* suppresses the tumor frequency (C; arrowheads) and (D) though some *hop*^*Tum-l*^*;;hipk*^*4*^*/TM6B* flies die in the early pupal stage, many reach the late pupal pharate adult stage. (E) Quantification of the *hop*^*Tum-l*^ lethal stages in (A-D); *hop*^*Tum-l*^*;; MKRS/TM6B* (blue; n = 148) and *hop*^*Tum-l*^*;; hipk*^*4*^*/TM6B* (orange; n = 193). (F-I) The *hop*^*Tum-l*^ tumor severity assay, performed at 25°C, involved phenotypically ranking flies into three categories: (F) represents ‘class 1’ where flies have greater than 5 tumors ranging in size from small to large, (G) represents ‘class 2’ where more than 5 small to medium tumors are present, and (H) represents ‘class 3’ where less than 5 small tumors are present. (I) Quantification of the *hop*^*Tum-l*^ tumor severity assay; *hop*^*Tum-l*^*/Y; hml-GAL4/+* (blue; n = 52), and *hop*^*Tum-l*^*/Y; hml-GAL4/UAS-hipk*^*RNAi*^ (orange; n = 48).

The lethality assay in Fig 1E was performed by crossing 50 females and 15 males from each stock (*hop*^*Tum-l*^*;; MKRS/TM6B* and *hop*^*Tum-l*^*;; hipk*^*4*^*/TM6B*) in a bottle and raising flies at 29°C. After 11 days, all pupae were removed from the walls of the bottles and were ranked as either ‘early pupal lethal’ (had no recognizable adult structures), ‘late pupal lethal’ (pharate adults), or ‘eclosed adult’ (see examples of each rank in S1 Fig). The tumor frequency assay in Fig 1F–1I was performed by crossing 8 females (*hop*^*Tum-l*^*/(FM7); hml-GAL4)* to 6 males (either *w*^*1118*^*/Y* or X/Y; *UAS-hipk*^*RNAi*^) in a vial and raising flies at 25°C. After 13 days, male progeny were scored into the following classifications: ‘class 1’ (flies had greater than 5 tumors ranging in size from small to large), ‘class 2’ (more than 5 small to medium tumors were present), and ‘class 3’ (less than 5 small tumors were present).

### Proximity Ligation Assay (PLA)

PLA was performed on L3 wing discs according to manufacturer’s instruction (Duolink PLA, Millipore Sigma), and as described previously [32] with the following exceptions: discs were fixed in 4% formaldehyde for 15 minutes and discs were blocked with 1% normal donkey serum in PBT. The following primary antibodies and corresponding PLA probes were used: rabbit anti-HA (1:2000; Cell Signaling) and PLA probe anti-rabbit Plus, mouse anti-MYC (1:200, ABM) and PLA probe anti-mouse Minus. A subset of discs was stained to validate HA-Hipk and Stat92E-MYC expression (S5 Fig).

## Results and discussion

### *hop*^*Tum-l*^-induced lethality is rescued by reducing *hipk*

Previous studies have shown that overexpression of Hipk induces melanotic tumors, a phenotype reminiscent of a dominant mutation in the Drosophila JAK homologue *hop* [21]. Extensive characterization of the *hop*^*Tum-l*^ allele by others has shown that it can be utilized in lethality and tumor frequency genetic interaction assays to identify novel JAK/STAT pathway components and regulators [10,33,34]. We tested whether *hipk* could modify the *hop*^*Tum-l*^ lethality phenotype. *hop*^*Tum-l*^ animals raised at 29°C were larval or early pupal lethal (Fig 1A and 1B); 91% of pupae died in the early pupal stage, 9% died in the late pupal stage, and 0% of adults eclosed (Fig 1E, for scoring classification, see S1 Fig). Heterozygous reduction of *hipk* using the *hipk*^*4*^ null allele [28] in *hop*^*Tum-l*^;; *hipk*^*4*^/TM6B animals suppressed the *hop*^*Tum-l*^ phenotype (Fig 1C and 1D); 23% of pupae died during early pupal development, 76% died as pharate adults and 1% were able to eclose (Fig 1E). Thus, we conclude that Hipk is a positive regulator of the pathway since reducing Hipk suppressed phenotypes caused by overactive JAK/STAT.

Since *hop*^*Tum-l*^ tumors derive from hemocytes [11,35], we asked whether reduction of Hipk within the hemocytes could rescue *hop*^*Tum-l*^ lethality at 29°C. We expressed UAS-*hipk* RNAi in hemocytes using *hml-GAL4* in a *hop*^*Tum-l*^ genetic background (*hop*^*Tum-l*^; *hml>hipk*^*RNAi*^*)* but did not observe a substantial suppression under these conditions. We reasoned that the mild rescue is possibly due to a combination of the strength of the *hop*^*Tum-l*^ phenotype at 29°C, and weakness of *hml>UAS-hipk*^*RNAi*^. *hop*^*Tum-l*^ is temperature sensitive, yielding a more severe phenotype at 29°C than at 25°C. We therefore tested whether loss of *hipk* within hemocytes could rescue the *hop*^*Tum-l*^ phenotype at 25°C. *hop*^*Tum-l*^*;hml-GAL4/+* flies raised at 25°C exhibited a range of tumor frequencies: 15% of flies had more than 5 small to large tumors (class 1; Fig 1F and 1I), 50% of flies had more than 5 small to medium tumors (class 2; Fig 1G and 1I), and 35% of flies had less than 5 small tumors (class 3; Fig 1H and 1I). Reducing *hipk* (*hop*^*Tum-l*^*; hml-GAL4/UAS-hipk-RNAi*) suppressed the severity of *hop*^*Tum-l*^ induced tumors. We observed 0% of flies in class 1, 29% of flies in class 2, and 71% of flies in class 3 (Fig 1I). The induced expression of various *hipk* transgenes has no significant effect on the tumor count in a *hop*^*Tum-l*^ sensitized background (S2 Fig). We conclude that *hipk* is required for the full severity of the *hop*^*Tum-l*^ phenotype.

### Hipk promotes JAK/STAT signalling, downstream of Upd

**Fig 2:**
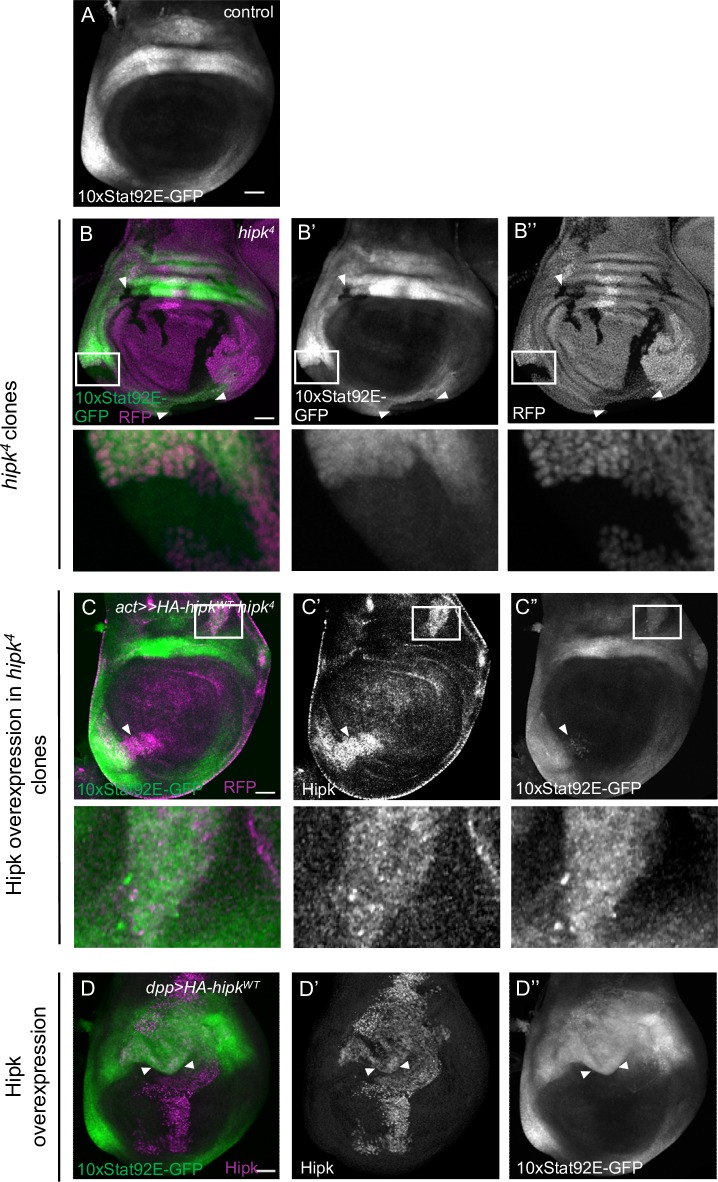
Hipk promotes endogenous JAK/STAT signaling. (A) A control L3 wing disc showing the expression domain of the transcriptional reporter *10xStat92E-GFP* which highlights location and extent of endogenous JAK/STAT signaling. (B-B”). Expression of *10xStat92E-GFP* is perturbed in *hipk*^*4*^ mutant somatic clones marked by the absence of RFP (arrowheads) (n = 20). (C-C”) Expressing *UAS-HA-hipk*^*WT*^ within *hipk*^*4*^ MARCM clones (marked with Hipk antibody staining) (*act>>HA-hipk*^*WT*^; *hipk*^*4*^) restores and can elevate *10xStat92E-GFP* levels (n = 10). (D-D”). Overexpression of Hipk in *dpp>HA-hipk*^*3M*^ wing discs causes elevated *10xStat92E-GFP* expression (arrowheads) (n = 20). Boxed regions in B-B” and C-C” mark zoomed-in regions in the lower panels.

Recent studies have shown that the JAK/STAT target gene *dMyc* is upregulated upon overexpression of *hipk* in wing imaginal discs [36]. We therefore asked if Hipk influences JAK/STAT activity more generally. We utilized the Stat92E-responsive transcriptional reporter *10xStat92E-GFP* in third instar larval (L3) imaginal discs, which provides an accurate representation of endogenous pathway activity [22](Fig 2A). Loss of *hipk* in somatic clones led to significant cell-autonomous reductions in *10xStat92E-GFP* expression by in wing imaginal discs (Fig 2A–2B”), indicating that Hipk is required for reporter gene expression. This effect can be rescued by expressing *HA-Hipk WT* within *hipk*^*4*^ clones, and in some instances results in up to 2.5 fold elevated *10xStat92E-GFP* levels within the clone compared to neighboring cells, suggesting that Hipk can induce reporter gene expression, and is sufficient for activation of the reporter gene expression (Fig 2C–2C”). To test this directly, we expressed Hipk at a high level in a stripe along the anterior-posterior axis in the wing disc using *dpp-Gal4* and growing the cross at 29°C which leads to enhanced Gal4 activity and overgrowth. We observed elevated *10xStat92E-GFP* expression in the expanded *dpp* expression domain, compared to control imaginal disc (arrow heads, Fig 2D–2D”). The induced reduction of *hipk* in the *dpp* domain by RNAi led to a significant reduction of another JAK/STAT target gene, *chinmo* [37], while overexpression of *Hipk WT* led to a marked increase in *chinmo* expression (S3A Fig). Previous studies have revealed that *chinmo* misexpression leads to tumor formation [38]. The reduced *chinmo* expression in *hipk*^*4*^ larvae may explain the reduced tumor phenotype seen in Fig 1.

Next, we asked if activity of JAK/STAT signaling is dependent on the localization of Hipk. We created two transgenic fly lines, UAS-HA-MYR-Hipk and UAS-HA-NLS-Hipk. The addition of a myristoylation tag MYR [29] leads Hipk to be localized on the cell membrane (S6B Fig). The addition of a nuclear localization signal NLS [30] leads to a defined localization of Hipk in nuclear speckles (S6C Fig). The induced expression of membrane bound Hipk (MYR-Hipk) led to an increase in expression of the transcriptional reporter *10xStat92E-GFP* while nuclear Hipk (NLS-Hipk) appears to have little to no effect (S3B Fig). Together, these results indicate that modulation of Hipk expression affects the level of JAK/STAT-dependent Stat92E activity in a cell-autonomous manner.

In vertebrates, the JAK/STAT signalling pathway can be activated by multiple cytokines and growth factors while in Drosophila, only three JAK/STAT ligands Upd, Upd2, and Upd3 were identified. The most potent of these is Upd [39]. We therefore focused further analyses on Upd only. Previous studies have shown that Upd controls eyes size through the JAK/STAT pathway, and disruption of *upd* causes a small eye phenotype [27](S4A and S4C Fig). Reduction of *hipk* also caused a mild to moderate small eye phenotype [18] (S4B Fig). While heterozygosity for *hipk*^*4*^ had no eye phenotype on its own, it significantly enhanced the small eye phenotype seen in *upd* (S4D and S4E Fig).

To determine whether Hipk promotes JAK/STAT activity by affecting expression of the ligand Upd, we examined *upd-lacZ* expression upon modulation of Hipk levels in L3 eye discs using *upd*^*1*^*-lacZ* [27] in *hipk*^*4*^ clones. *upd* is expressed in cells at the posterior center of the L3 eye-antennal disc (S4F Fig). If Hipk promoted Stat92E-dependent gene expression by inducing *upd* expression, we would expect loss of *hipk* to result in reductions of *upd*. In contrast, we found that loss of *hipk* in the eye disc causes a slight upregulation of *upd-*lacZ reporter expression (S4G Fig). We conclude that Hipk likely promotes JAK/STAT activity downstream of Upd.

### Hipk interacts with Stat92E

Previously, we have shown that Hipk induces overproliferation and invasive cell behaviour, and established an *in vivo* model to test components of various signalling pathways for their ability to suppress this Hipk-mediated phenotype in L3 wing discs [21]. We have shown that the individual knockdown of JAK/STAT pathway components cannot suppress *hipk* induced overgrowth, and that overexpression of Stat92E alone does not phenocopy the *hipk* overexpression phenotypes [21].

**Fig 3:**
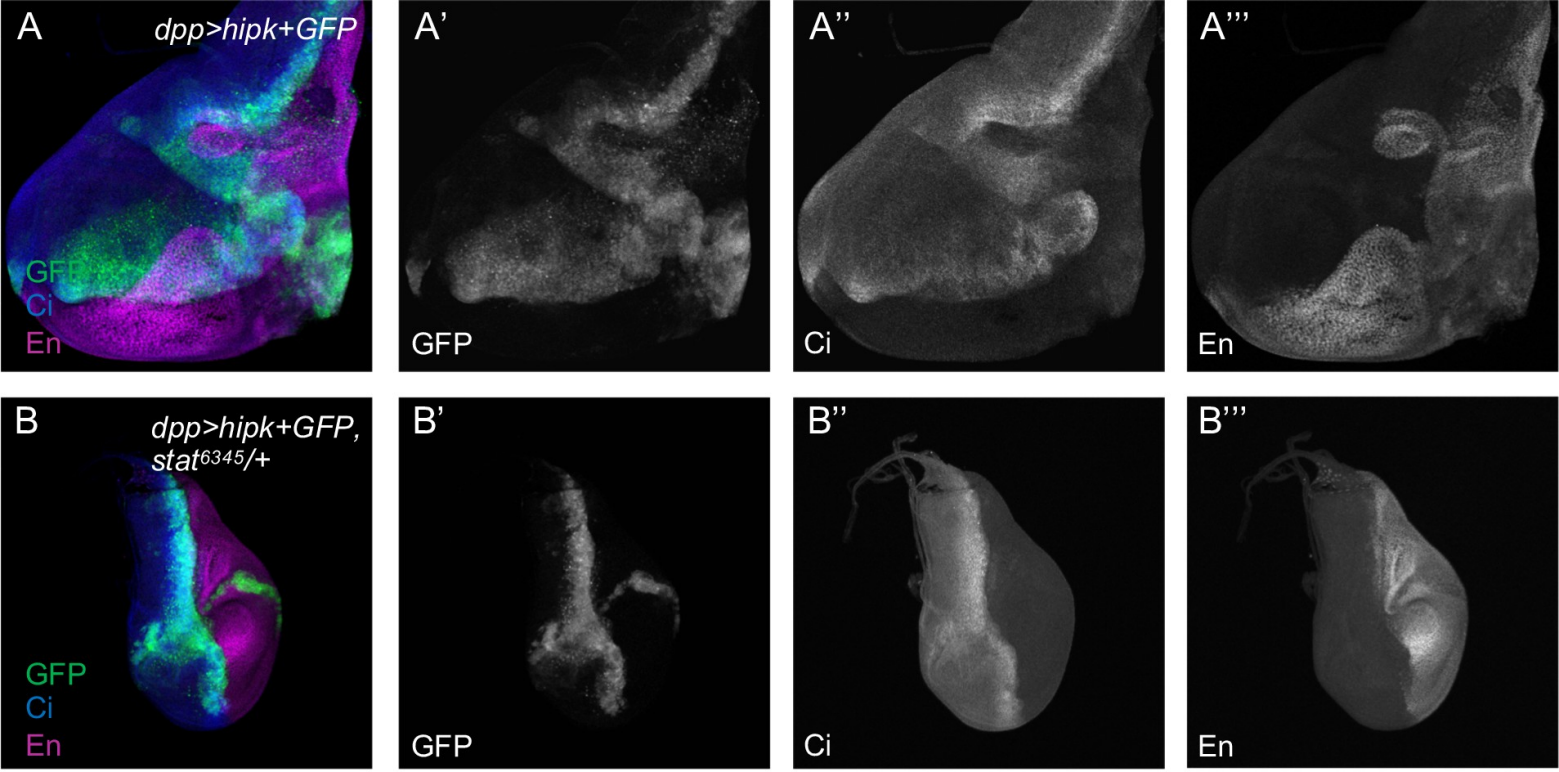
Reduced Stat92E partially suppresses Hipk-induced cell spreading. (A-A”’) An L3 wing disc from *dpp>HA-hipk*^*1M*^*+GFP* showing Hipk-induced overproliferation and cell spreading of GFP-positive Hipk expressing cells. (B-B”’) Reduction of Stat92E in *dpp>HA-hipk*^*1M*^*+GFP*, *stat*^*6346/+*^ discs can rescue the overgrowth phenotype, and partially rescue cell spreading. Anti-Ci (blue) and anti-Engrailed (En, mangenta) are used to label the anterior and posterior compartments, respectively. GFP (green) marks the anterior-posterior *dpp* expression domain. All larvae were raised at 29°C (n = 5).

When using two copies of *hipk* in *dpp>2xhipk+ GFP*, we observed the same overgrowth of L3 wing discs (Fig 3A). Using anterior and posterior markers (Cubitus interruptus Ci, and Engrailed En, respectively), we observed GFP+ cells outside of the *dpp* domain, in the anterior and posterior domains, confirming that Hipk induces cell spreading (Fig 3A–3A”’). When one copy of Stat92E is removed using the amorphic allele *stat*^*06346*^ [40], the Hipk induced overgrowth can be rescued. Cell spreading still occurs but to a lesser extent (Fig 3B–3B”’). This implies a genetic interaction of Hipk with Stat92E.

**Fig 4:**
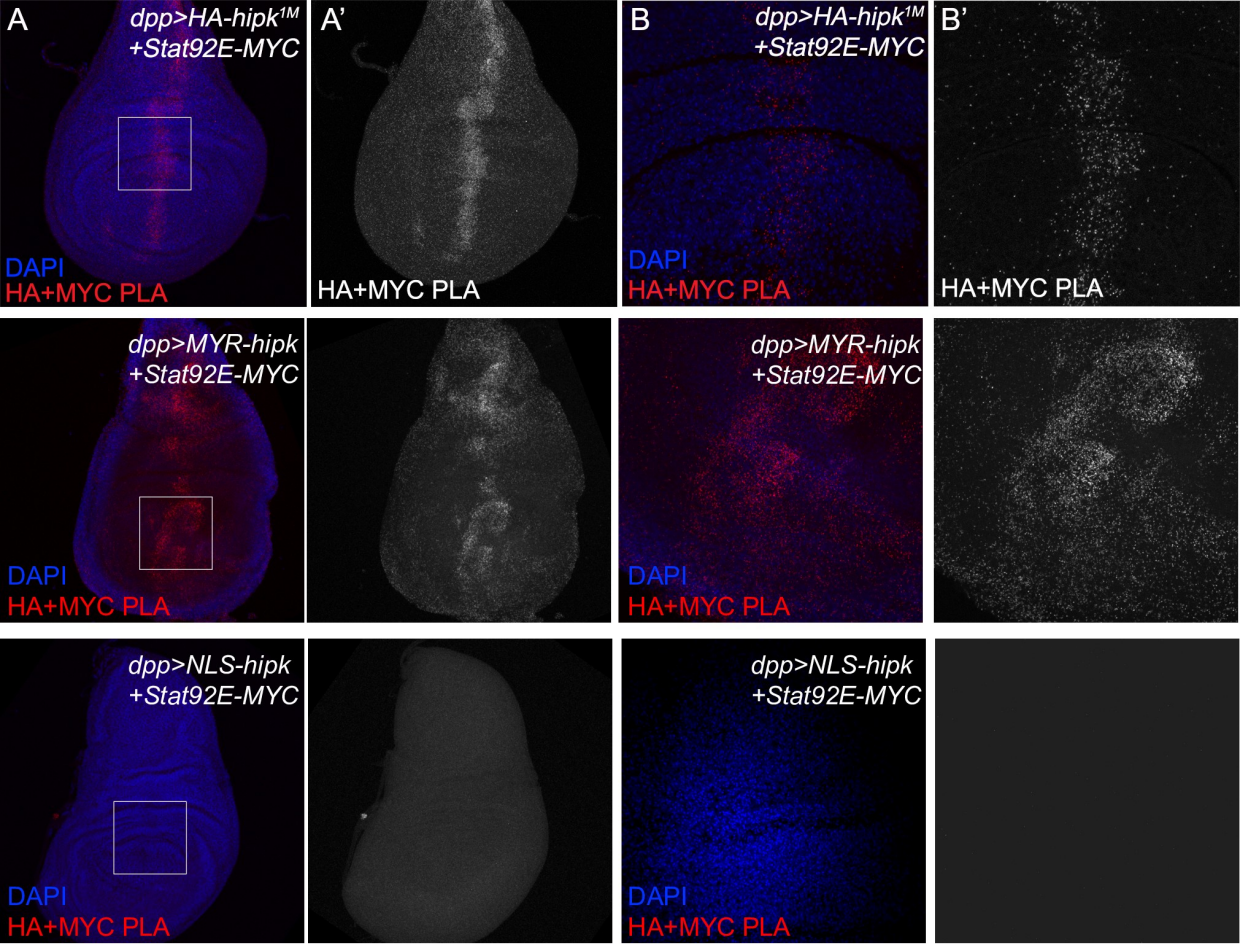
HA-Hipk and Stat92E-MYC physically interact in the wing imaginal disc. Proximity Ligation Assays (PLA) were performed on L3 wing imaginal discs by probing with antibodies against HA and MYC tags to detect HA-Hipk and Stat92E-MYC, respectively. A positive PLA signal is observed along the *dpp* domain in (A, B) *dpp>HA-hipk*^*1M*^*+Stat92E-MYC* and (C,D) *dpp>MYR-HA-hipk+Stat92E-MYC* discs, but not in (E,F) *dpp>NLS-HA-hipk+Stat92E-MYC* discs. Boxed regions in A, C, E represent zoomed-in regions in B, D, F. Images in C, D represent a membrane focal plane, and thus exclude the DAPI stained nuclei. Scale bars equal 10μm.

Hipk primarily localizes to the nucleus and can also be seen in the cytoplasm [41]. Little is known about Hipk functions outside of the nucleus. Because Stat92E is also found in the nucleus we began testing for a physical Hipk-Stat92E interaction. We utilized a proximity ligation assay (PLA), which can detect whether two proteins of interest are less than 40nm apart *in vivo* [42]. We probed *dpp>HA-hipk*^*1M*^*+Stat92E-MYC* wing discs with anti-HA and anti-MYC antibodies and observed a positive PLA reaction (Fig 4A–4B’). To minimize the effects of excessive protein expression in these cells, we expressed transgenes at levels that did not induce any aberrant phenotypes. Thus, the interaction is unlikely to be due to protein saturation. Negative control discs *(dpp*>*HA-hipk*^*1M*^*+GFP*) that were probed against GFP and HA did not yield a PLA signal (S5 Fig).

To further characterize the subcellular localization of this interaction, we utilized UAS-MYR-HA-Hipk and UAS-NLS-HA-Hipk transgenic flies. In a PLA assay using *dpp*>*MYR-HA-Hipk+Stat92E-MYC* wing discs, we observed clear positive signals indicating a physical interaction of membrane associated Hipk with Stat92E (Fig 4C–4D’). We tested for an interaction using *dpp*>*NLS-HA-Hipk*+*Stat92E-MYC* wing discs. Surprisingly, we did not observe a PLA signal between NLS-Hipk and Stat92E (Fig 4E–4F’). Collectively, these data suggest that Hipk and Stat92E physically interact in wing disc cells, and that this occurs at or near the cell membrane, which is where Stat92E interacts with Hop and Dome [4].

### Hipk can modulate Stat92E localization

To support these findings, we examined the subcellular localization of exogenous GFP-tagged Stat92E (Stat92E-GFP [26]) and Hipk in salivary glands. The large size of salivary gland cells provides an ideal system for subcellular localization studies. Previous studies described the localization of Stat92E as both nuclear and cytoplasmic, as well as membrane associated [25,43,44]. In *dpp*>*lacZ+* Stat92E-GFP L3 salivary glands, Stat92E is distributed diffusely throughout the cytoplasm, and accumulates in the nucleus and on the membrane (S6D Fig). Both HA-Hipk and NLS-HA-Hipk primarily show a nuclear speckle localization, while MYR-HA-Hipk is found primarily at the cell cortex, and absent from the nucleus (S6 Fig).

**Fig 5:**
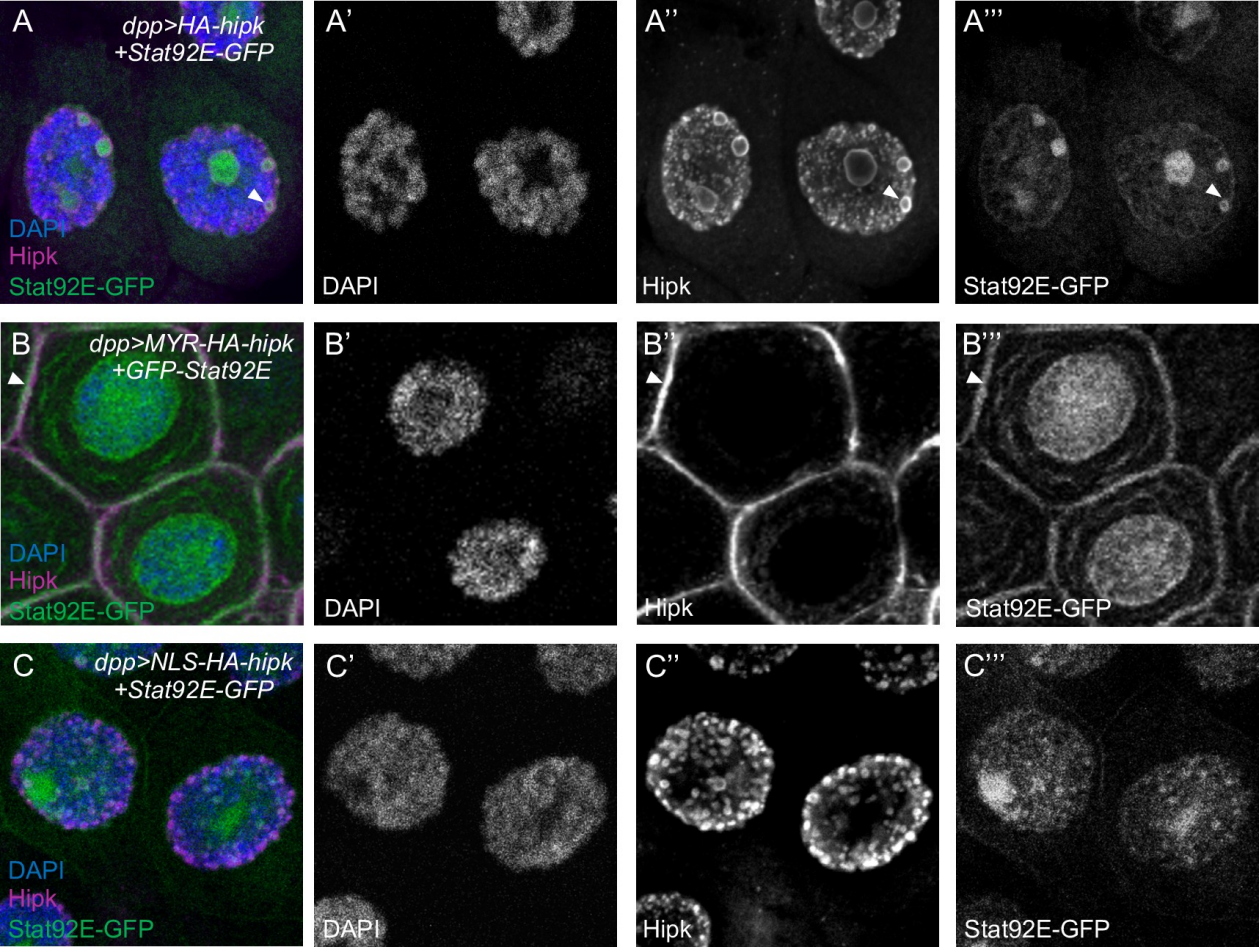
Hipk colocalizes with Stat92E in salivary gland cells. (A-A”’) *dpp-Gal4* which is expressed in salivary gland cells, was used to express Hipk and Stat92E transgenes. HA-Hipk^1M^ and GFP-Stat92E partially colocalize in nuclear speckles. (B-B”’) MYR-Hipk and GFP-Stat92E co-localize on the cell membrane. (C-C”’) NLS-Hipk and GFP-Stat92E do not colocalize. Nuclei were stained with DAPI (blue), HA-Hipk is probed with anti-dHipk (magenta), Stat92E-GFP is green. All larvae were raised at 29°C. Individual sections rather than maximum projections are shown to accurately represent the presence or absence of co-localization.

The co-expression of Stat92E-GFP and HA-Hipk leads to a translocation of Stat92E-GFP such that Stat92E localizes mainly in the nucleus and can also be found in nuclear speckles where it partially co-localizes with HA-Hipk (Fig 5A–5A”’). Co-expression of a MYR-tagged Hipk and Stat92E-GFP in L3 salivary glands reveals a co-localization of both proteins near the cell membrane (Fig 5B–5B”’), and Stat92E is now strongly enriched at the membrane. The addition of an NLS to Hipk caused a defined localization in small nuclear speckles. However, Stat92E-GFP does not co-localize in these small speckles. Stat92E-GFP remains mainly nuclear in larger speckles, with faint membrane localization (Fig 5C–5C”’). These studies support the findings from the PLA interaction studies and suggest that non-nuclear Hipk interacts with Stat92E.

## Conclusions

**Fig 6:**
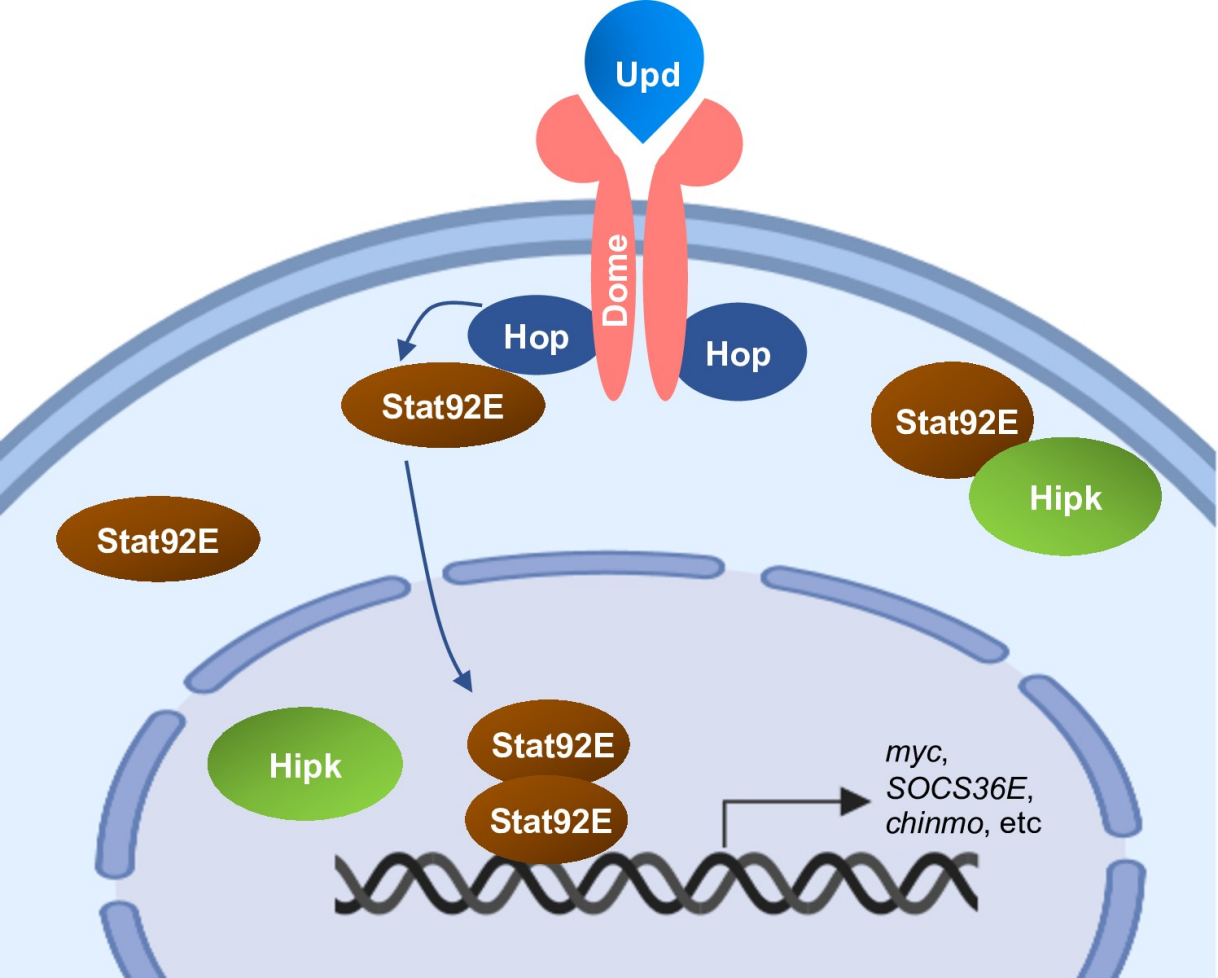
Hipk is a regulator of the JAK/STAT pathway. Stat92E and Hipk interact at or near the plasma membrane. Hipk affects Stat92E localization and is required for pathway activity which leads to misexpression of JAK/STAT target genes.

In summary, we present novel evidence that Hipk is a regulator of the JAK/STAT pathway and acts downstream of Upd. The transcriptional output of JAK/STAT signaling is perturbed upon loss of *hipk*, and conversely increased Hipk induces elevated JAK/STAT activity in a cell autonomous manner. Genetic interaction studies reveal that Hipk is required for the full potency of the *hop*^*Tum-l*^ allele. Further, we provide *in vivo* data that suggests a physical interaction between Hipk and Stat92E. Given that membrane-associated Hipk has the ability to re-localize Stat92E, we propose that they interact inside the cell under physiological conditions and that the interaction is necessary for Stat92E activity (Fig 6). It is possible that Hipk can modulate the ability of Stat92E to interact with Dome and Hop at the membrane, and future studies could investigate this mechanism.

Our work is consistent with previous reports investigating vertebrate Hipks in which they found that an activated version of Hipk2 phosphorylates Stat3 and promotes its nuclear activities [45,46]. Furthermore, Hipk2 has been identified as a potential drug target in treating Acute Myeloid Leukemia, which is due to activated JAK/STAT signaling [47]. Future studies with help determine the precise mechanism of Hipk’s role in this pathway and could ultimately lead to new therapeutics used to treat human cancers.

## Supporting information

S1 Fig Scoring classification for phenotypical analyses in Fig 1. The *hop*^*Tum-l*^ lethality assay was ranked into three categories: (A) represents category ‘early pupal lethal’, where no adult structures are detectable, (B) represents ‘late pupal lethal’, where adult structures are visible but the fly does not eclose, and (C) represents the class ‘eclosed adults.
(PDF)

S2 Fig Quantification of *hop^Tum-l^* induced tumors after Hipk overexpression. The *hop*^*Tum-l*^ tumor severity assay was performed and classified as described for Fig 1. *hop*^*Tum-l*^ /Y; *hml-Gal4*,*UAS-GFP/UAS-lacZ* (Class 1: 36%, Class 2: 33%, Class 3: 31%, n = 139); *hop*^*Tum-l*^ /Y; *hml-Gal4*,*UAS-GFP/UAS-hipk*^*RNAi*^ (Class 1: 10%, Class 2: 18%, Class 3: 72%, n = 61); *hop*^*Tum-l*^ /Y; *hml-Gal4*,*UAS-GFP/UAS-HA-Hipk* (Class 1: 38%, Class 2: 42%, Class 3: 20%, n = 91); *hop*^*Tum-l*^ /Y; *hml-Gal4*,*UAS-GFP/UAS-MYR-HA-Hipk* (Class 1: 35%, Class 2: 36%, Class 3: 29%, n = 98); *hop*^*Tum-l*^ /Y; *hml-Gal4*,*UAS-GFP/UAS-NLS-HA-Hipk* (Class 1: 30%, Class 2: 41%, Class 3: 29%, n = 76).
(PDF)

S3 Fig Quantification of JAK/STAT activity. (A) Reduction of *hipk* in *hipk*^*4*^ L3 larvae (*dpp>hipk*^*RNAi*^) leads to reduction of *chinmo* expression while overexpression of *Hipk* (*dpp>HA-Hipk*) leads to an increase. (B) Reduction of *hipk* in *hipk*^*4*^ clones (*act>RFP*, *hipk*^*4*^,*10xStat92E-GFP*) leads to a decrease of *10xStat92E-GFP* expression, compared to neighbouring wild-type cells (n = 3) while overexpression of *Hipk* in *hipk*^*4*^ clones (*act>RFP*, *hipkWT*, *hipk*^*4*^,*10xStat92E-GFP*) leads to a rescue and slight increase of the reporter gene expression (n = 5). Overexpression of membrane bound *Hipk* (MYR-Hipk) in *hipk*^*4*^ clones (*act>RFP*, *MYR-hipk*, *hipk*^*4*^,*10xStat92E-GFP*) can also rescue and cause a slight increase (n = 5), while overexpression of nuclear *Hipk* in *hipk*^*4*^ clones (*act>RFP*, *NLS-hipk*, *hipk*^*4*^,*10xStat92E-GFP*) can rescue but does not cause an increase in reporter gene expression (n = 6). Total RNA from L3 larval heads was extracted using RNeasy Mini Kits. First strand cDNA was synthesized using OneScript Plus cDNA Synthesis Kit. qRT-PCR were performed using SensiFast SYBR Lo-ROX Kit on QuantStudio3 Real Time PCR System (ThermoFisher).
(PDF)

S4 Fig Hipk promotes JAK/STAT signaling, downstream of Upd. (A-D) Adult eyes of the indicated genotypes are shown. (A) *TM3/TM6B* as control. Loss of hipk (*hipk*^*4*^*/TM6B*, *B*), and loss of *os* (*upd*) (C), lead to a small eye. (D) This phenotype is significantly enhanced in *os;;hipk*^*4*^*/TM6B* flies, P<0.0001. (E) Quantification of eye area for flies shown in (A-D), n = 10 for each group. (F-G”) Loss of *hipk* does not affect *upd-lacZ*. (F) *upd-lacZ* is expressed at the posterior center of the L3 eye-antennal control disc. (G-G”) Loss of *hipk*, in negatively marked RFP clones, does not alter *upd-lacZ* expression (arrowhead) (n = 20). Scale bars equal 10μm. 10 images were acquired for *TM3/TM6B*, *hipk*^*4*^*/TM6B*, *os;;MKRS/TM6B*, and *os;;hipk*^*4*^*/TM6B* adult eyes. The area of each eye was measured in pixels using Photoshop, and the values were subjected to a student’s t-test
(PDF)

S5 Fig Negative controls for PLA, and expression of transgenes HA-Hipk and Stat92E-MYC in Fig 4. (A-A”) PLAs were performed on L3 wing imaginal discs by probing with antibodies against HA tag and GFP. There is no PLA signal detected between HA and GFP. (B-D) The various Hipk constructs are expressed in the dpp domain of L3 wing discs, and expression is verified by staining against HA tag. Image S5A” is overexposed to show outline of the disc and a clear absence of any PLA signal. (E) Stat92E-MYC is expressed in the dpp domain of L3 wing discs, expression is verified by staining against the MYC tag.
(PDF)

S6 Fig Subcellular localization of HA-Hipk and Stat92E-GFP in salivary gland cells of L3 larvae. (A-C) Expression of Hipk transgenes driven by sgs-Gal4 is verified by staining against Hipk. (A-A”) Hipk (red) is localized in the nucleus (DAPI-blue). (B-B”) MYR-Hipk is not detectable in the nucleus, localized throughout the cytoplasm and on the membrane. (C-C”) NLS-Hipk is localized in the nucleus. (D-D”) Expression of Stat92E-GFP (green) driven by dpp-Gal4 is membrane bound, cytoplasmic and nuclear. All larvae were raised at 29°C.
(PDF)
