# Supplementary figures and images for: Hipk is required for JAK/STAT activity during development and tumorigenesis
Source: PLoS One. 2019 Dec 31;14(12):e0226856. doi: 10.1371/journal.pone.0226856 (PMC6938406; doi:10.1371/journal.pone.0226856)

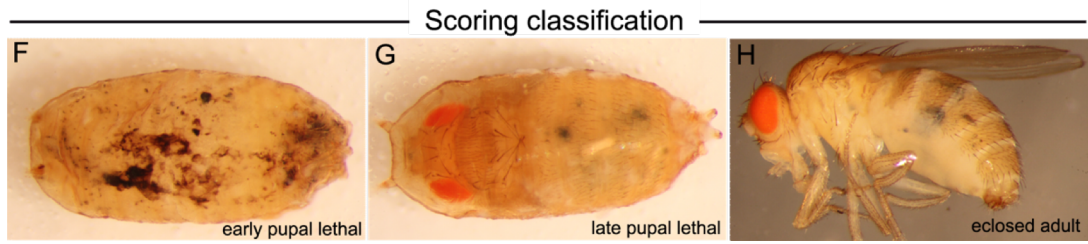

Figure S1

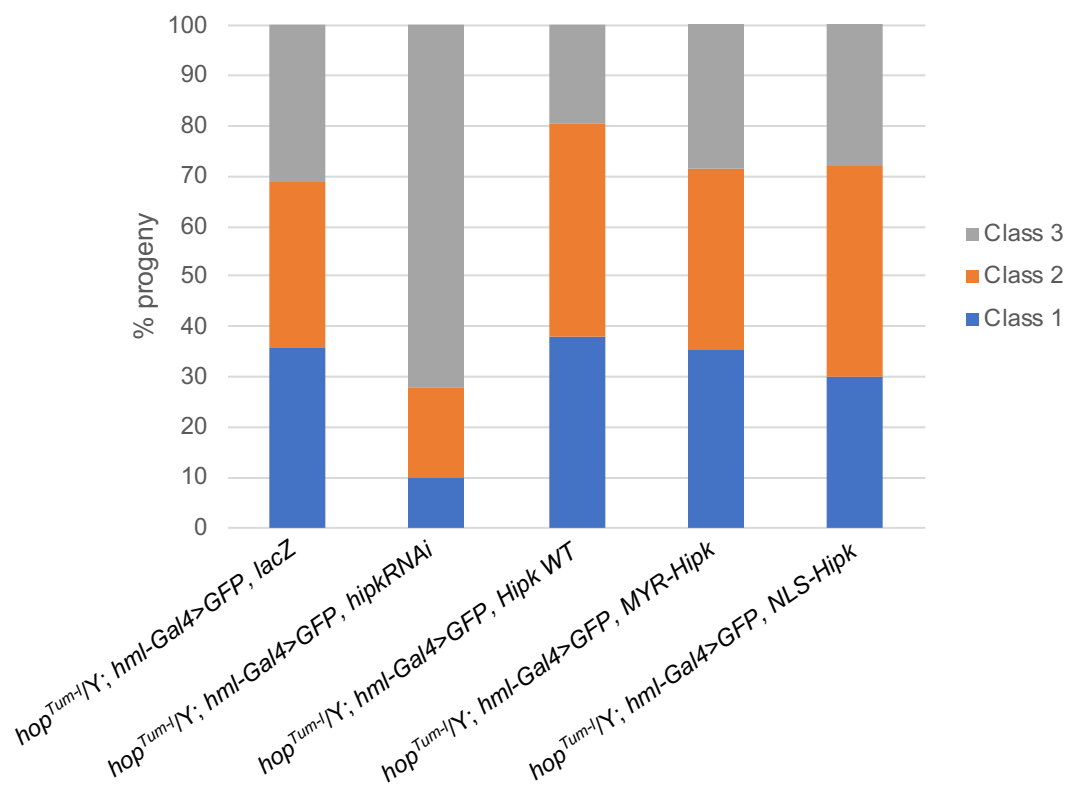

Figure S2

**A**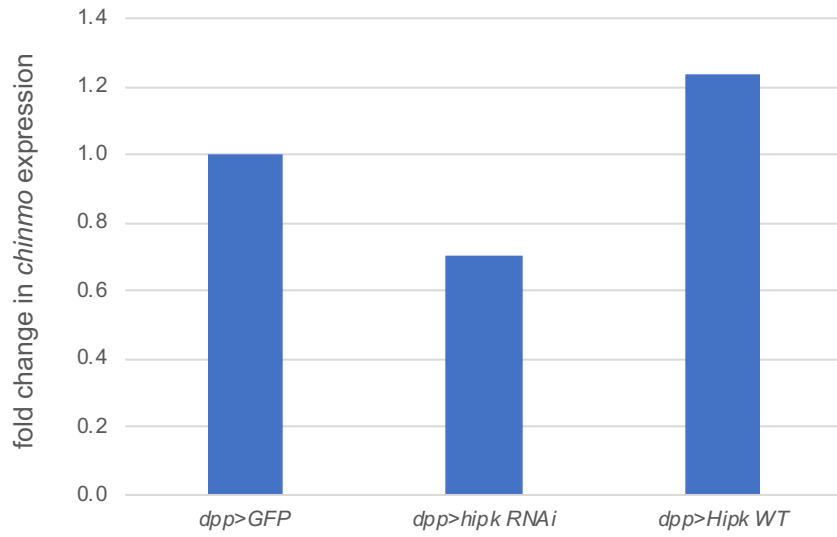**B**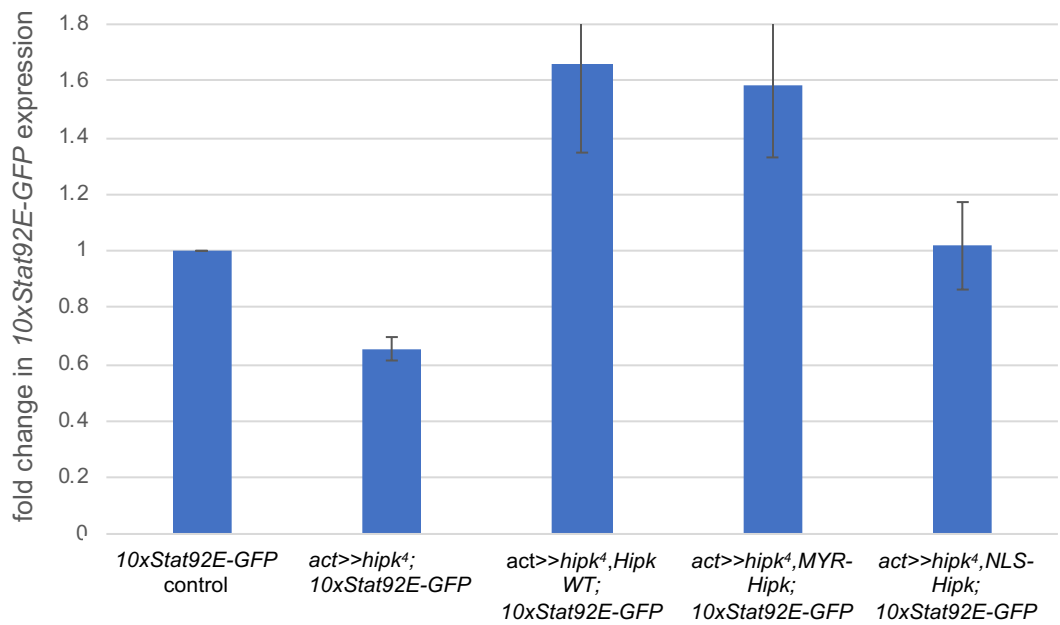

Figure S3

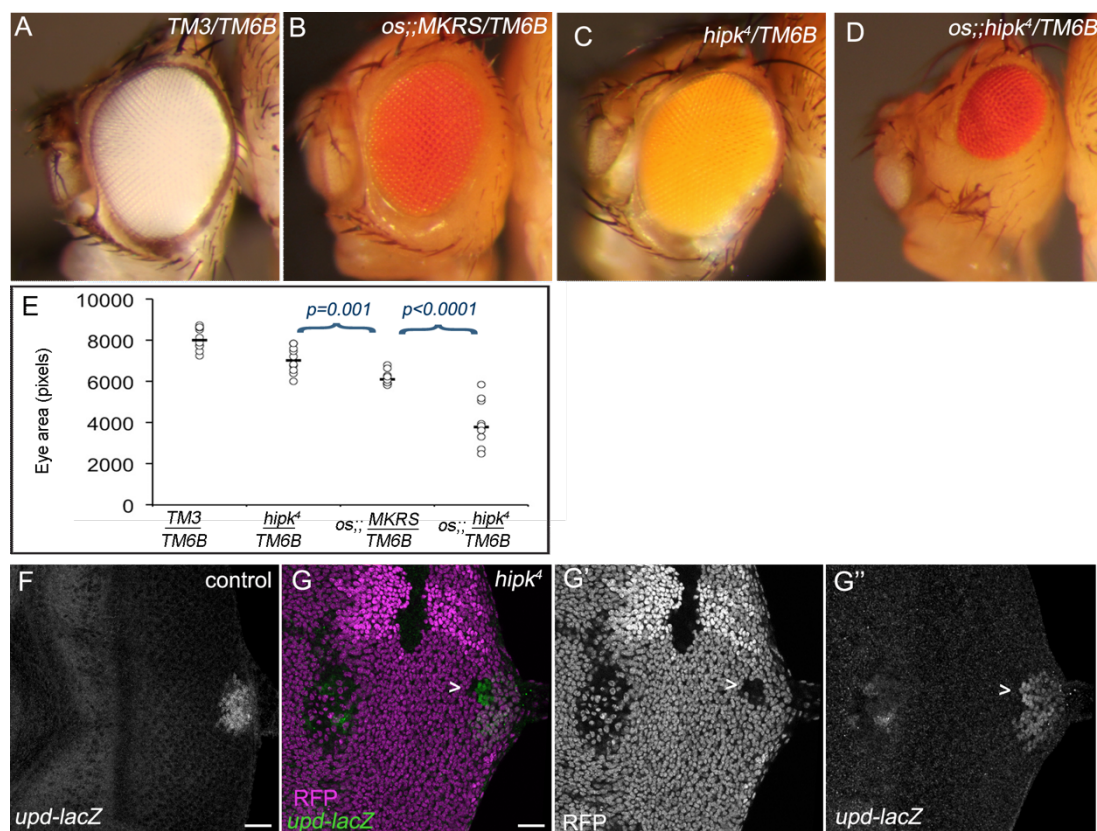

Figure S4

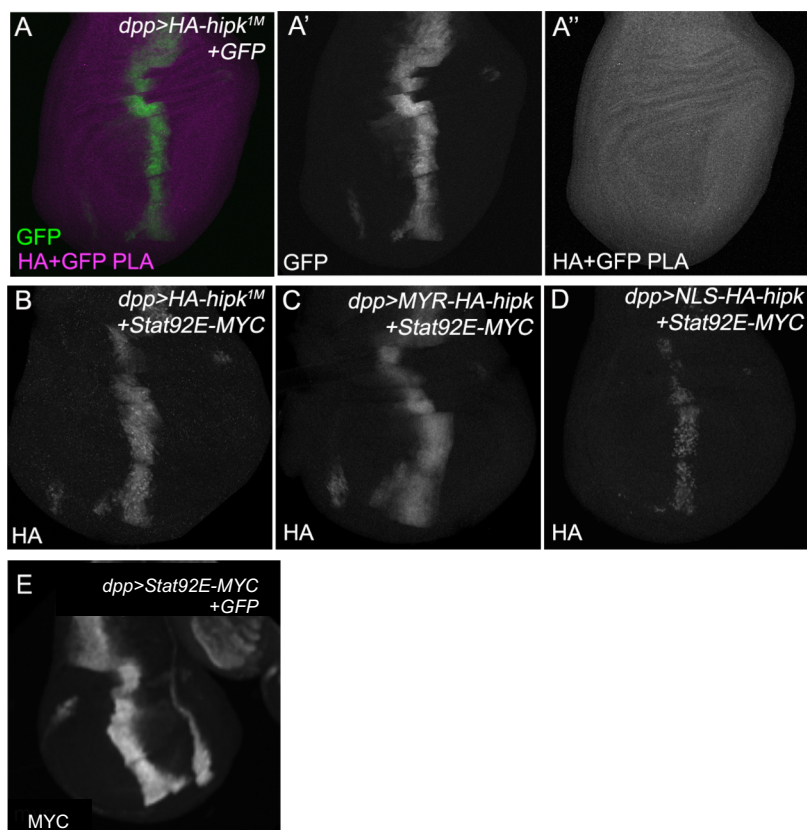

Figure S5

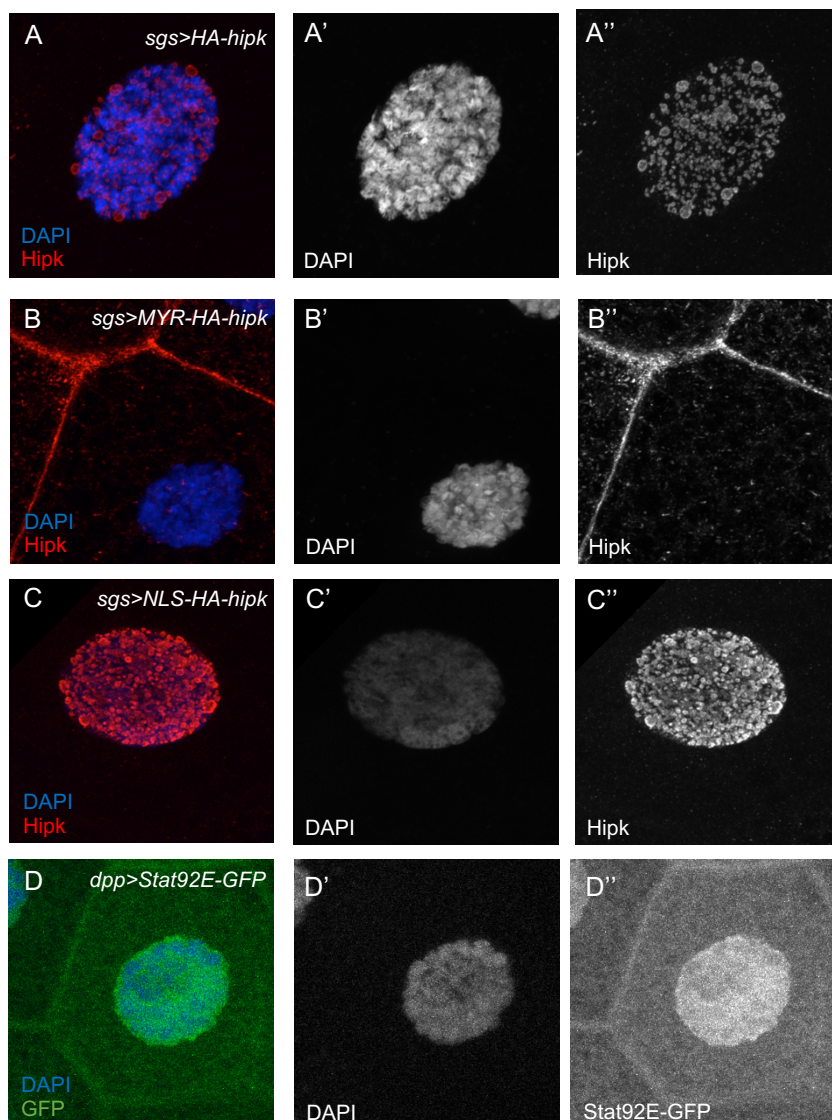

Figure S6

Supplement: S1 Fig — The hopTum-l lethality assay was ranked into three categories: (A) represents category ‘early pupal lethal’, where no adult structures are detectable, (B) represents ‘late pupal lethal’, where adult structures are visible but the fly does not eclose, and (C) represents the class ‘eclosed adults. (PDF) [file pone.0226856.s001.pdf]

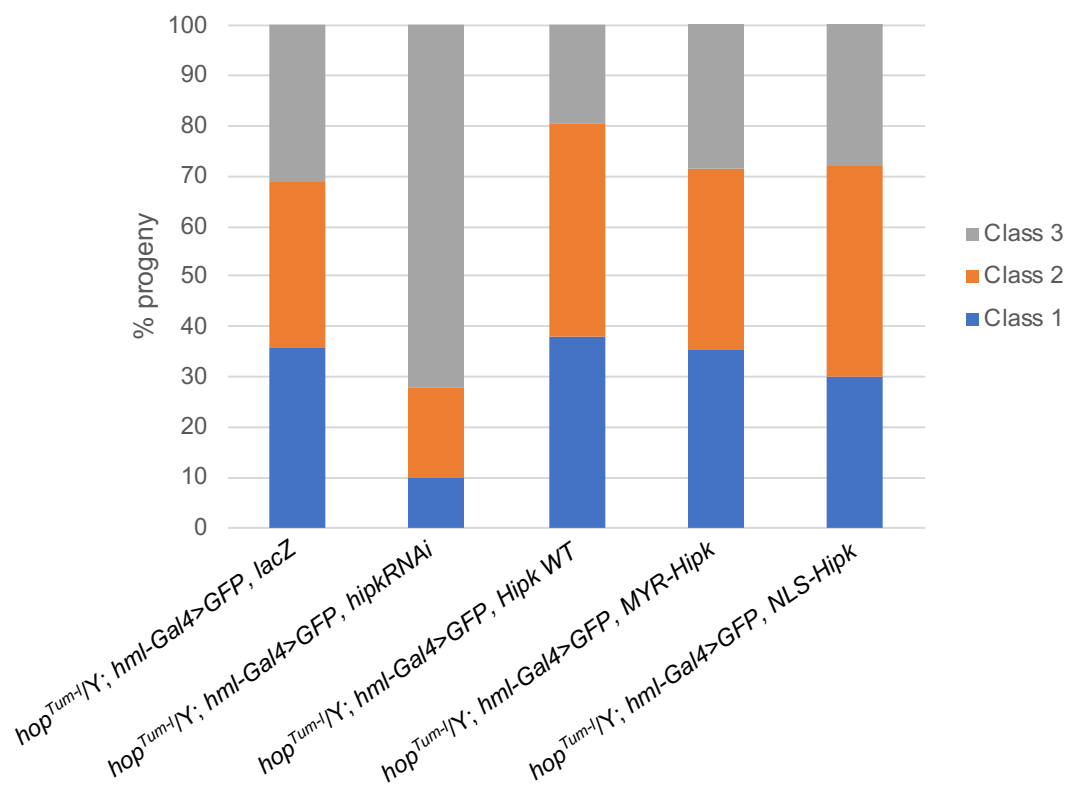

Figure S2

Supplement: S2 Fig — The hopTum-l tumor severity assay was performed and classified as described for Fig 1. hopTum-l /Y; hml-Gal4,UAS-GFP/UAS-lacZ (Class 1: 36%, Class 2: 33%, Class 3: 31%, n = 139); hopTum-l /Y; hml-Gal4,UAS-GFP/UAS-hipkRNAi (Class 1: 10%, Class 2: 18%, Class 3: 72%, n = 61); hopTum-l /Y; hml-Gal4,UAS-GFP/UAS-HA-Hipk (Class 1: 38%, Class 2: 42%, Class 3: 20%, n = 91); hopTum-l /Y; hml-Gal4,UAS-GFP/UAS-MYR-HA-Hipk (Class 1: 35%, Class 2: 36%, Class 3: 29%, n = 98); hopTum-l /Y; hml-Gal4,UAS-GFP/UAS-NLS-HA-Hipk (Class 1: 30%, Class 2: 41%, Class 3: 29%, n = 76). (PDF) [file pone.0226856.s002.pdf]

**A**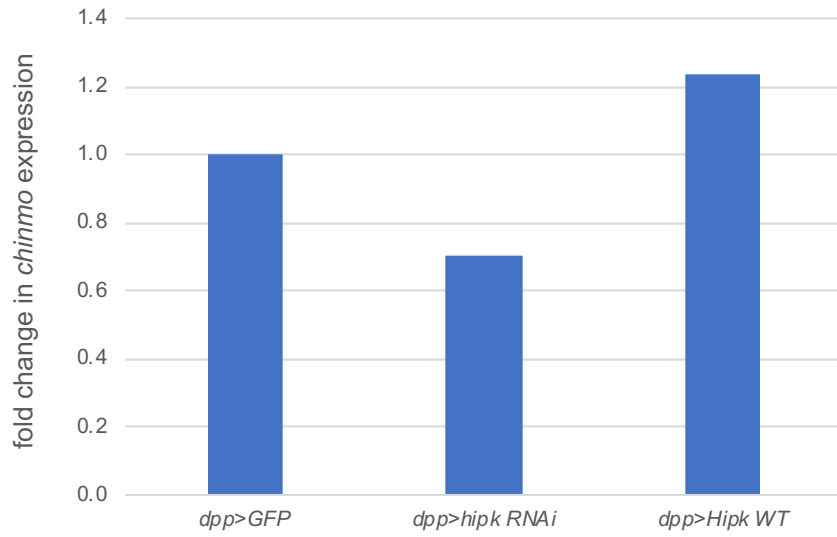**B**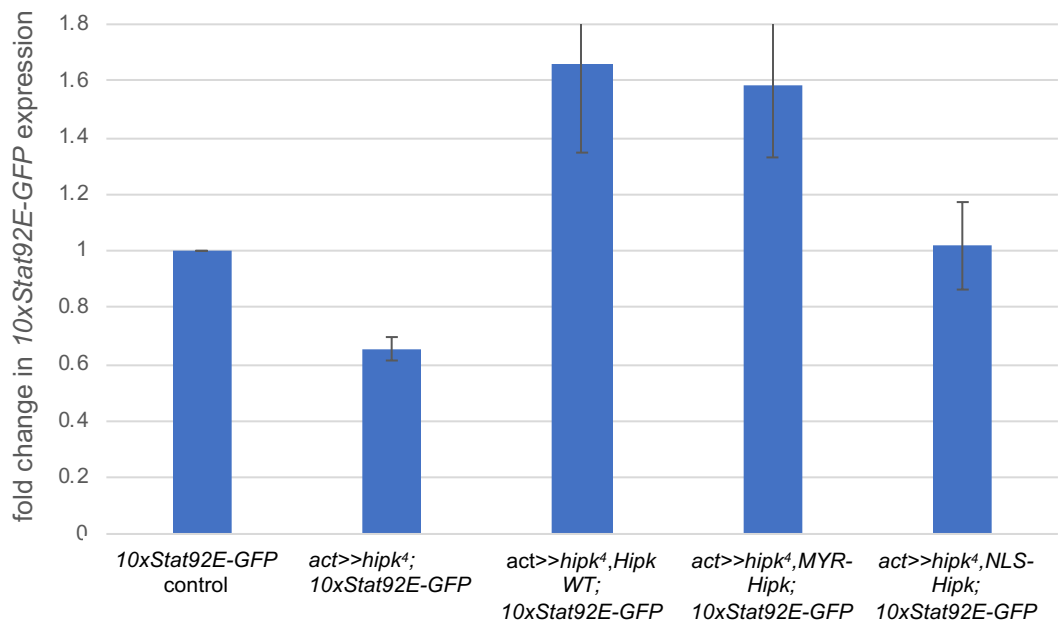

Figure S3

Supplement: S3 Fig — (A) Reduction of hipk in hipk4 L3 larvae (dpp>hipkRNAi) leads to reduction of chinmo expression while overexpression of Hipk (dpp>HA-Hipk) leads to an increase. (B) Reduction of hipk in hipk4 clones (act>RFP, hipk4,10xStat92E-GFP) leads to a decrease of 10xStat92E-GFP expression, compared to neighbouring wild-type cells (n = 3) while overexpression of Hipk in hipk4 clones (act>RFP, hipkWT, hipk4,10xStat92E-GFP) leads to a rescue and slight increase of the reporter gene expression (n = 5). Overexpression of membrane bound Hipk (MYR-Hipk) in hipk4 clones (act>RFP, MYR-hipk, hipk4,10xStat92E-GFP) can also rescue and cause a slight increase (n = 5), while overexpression of nuclear Hipk in hipk4 clones (act>RFP, NLS-hipk, hipk4,10xStat92E-GFP) can rescue but does not cause an increase in reporter gene expression (n = 6). Total RNA from L3 larval heads was extracted using RNeasy Mini Kits. First strand cDNA was synthesized using OneScript Plus cDNA Synthesis Kit. qRT-PCR were performed using SensiFast SYBR Lo-ROX Kit on QuantStudio3 Real Time PCR System (ThermoFisher). (PDF) [file pone.0226856.s003.pdf]

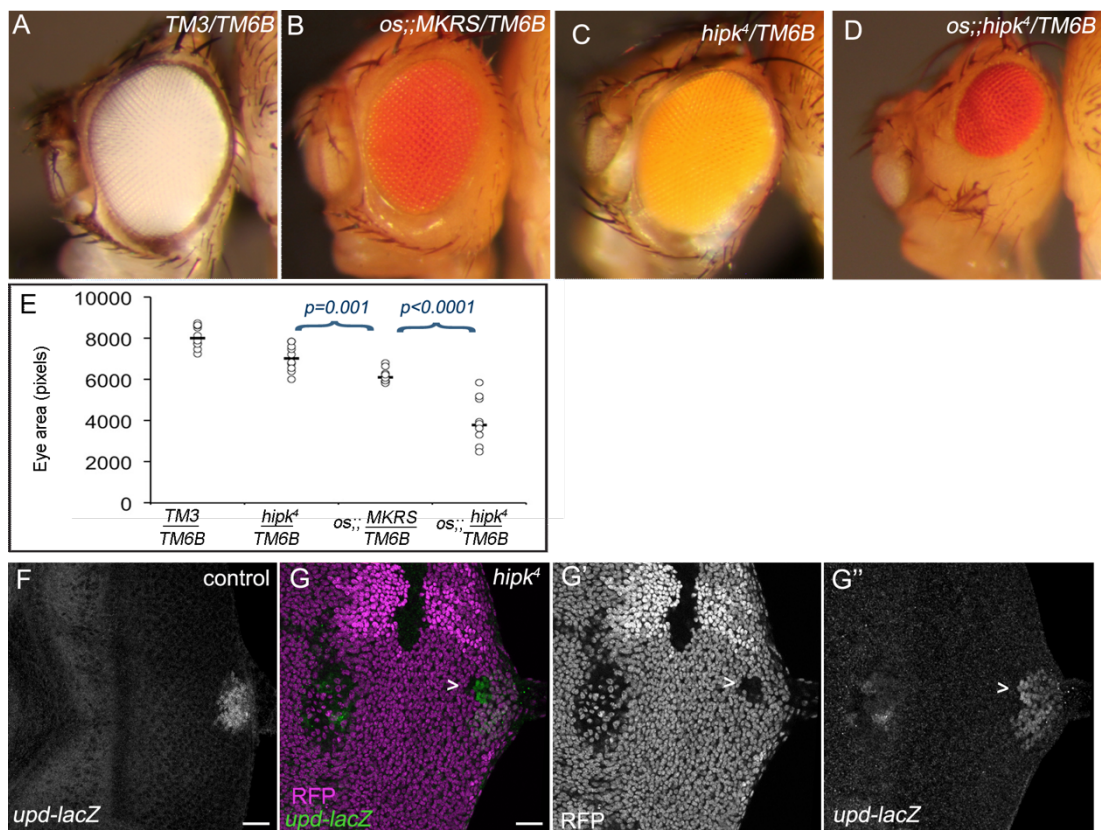

Figure S4

Supplement: S4 Fig — (A-D) Adult eyes of the indicated genotypes are shown. (A) TM3/TM6B as control. Loss of hipk (hipk4/TM6B, B), and loss of os (upd) (C), lead to a small eye. (D) This phenotype is significantly enhanced in os;;hipk4/TM6B flies, P<0.0001. (E) Quantification of eye area for flies shown in (A-D), n = 10 for each group. (F-G”) Loss of hipk does not affect upd-lacZ. (F) upd-lacZ is expressed at the posterior center of the L3 eye-antennal control disc. (G-G”) Loss of hipk, in negatively marked RFP clones, does not alter upd-lacZ expression (arrowhead) (n = 20). Scale bars equal 10μm. 10 images were acquired for TM3/TM6B, hipk4/TM6B, os;;MKRS/TM6B, and os;;hipk4/TM6B adult eyes. The area of each eye was measured in pixels using Photoshop, and the values were subjected to a student’s t-test (PDF) [file pone.0226856.s004.pdf]

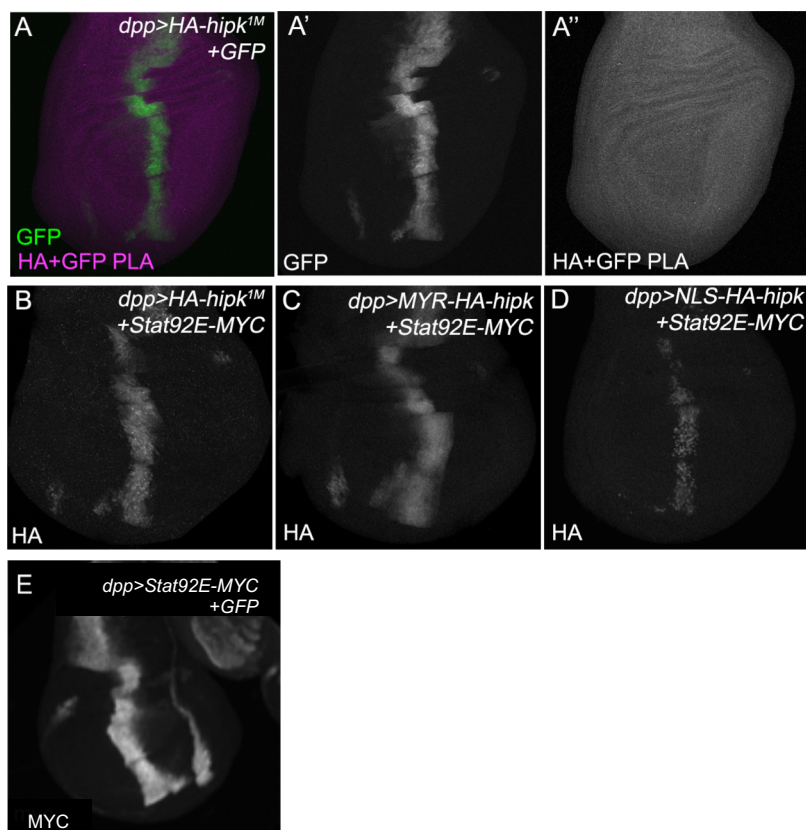

Figure S5

Supplement: S5 Fig — (A-A”) PLAs were performed on L3 wing imaginal discs by probing with antibodies against HA tag and GFP. There is no PLA signal detected between HA and GFP. (B-D) The various Hipk constructs are expressed in the dpp domain of L3 wing discs, and expression is verified by staining against HA tag. Image S5A” is overexposed to show outline of the disc and a clear absence of any PLA signal. (E) Stat92E-MYC is expressed in the dpp domain of L3 wing discs, expression is verified by staining against the MYC tag. (PDF) [file pone.0226856.s005.pdf]

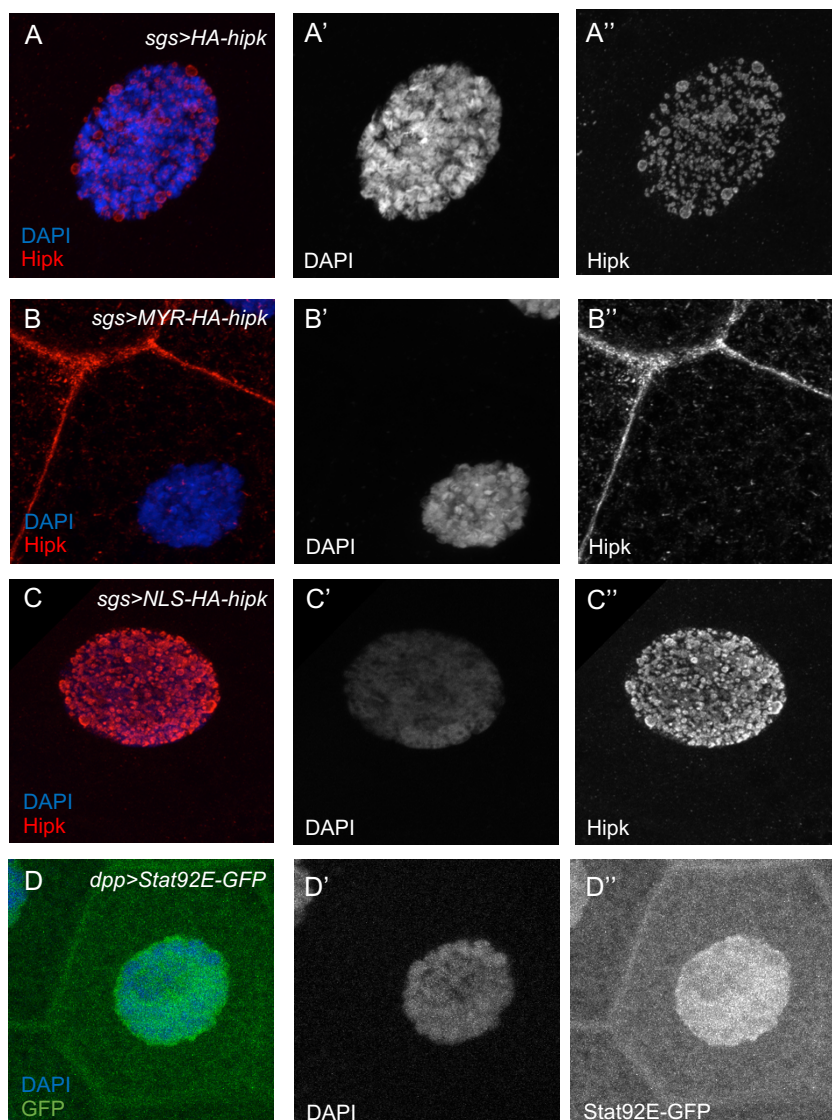

Figure S6

Supplement: S6 Fig — (A-C) Expression of Hipk transgenes driven by sgs-Gal4 is verified by staining against Hipk. (A-A”) Hipk (red) is localized in the nucleus (DAPI-blue). (B-B”) MYR-Hipk is not detectable in the nucleus, localized throughout the cytoplasm and on the membrane. (C-C”) NLS-Hipk is localized in the nucleus. (D-D”) Expression of Stat92E-GFP (green) driven by dpp-Gal4 is membrane bound, cytoplasmic and nuclear. All larvae were raised at 29°C. (PDF) [file pone.0226856.s006.pdf]
